# First real-world implementation of video-based emergency calls (VEC) in Germany: from vision to reality

**DOI:** 10.1016/j.resplu.2026.101398

**Published:** 2026-06-29

**Authors:** Henning Schmidtpott, Felix Reiser, Erik Lachfeld, Sebastian Heinrich, Julian Ganter

**Affiliations:** a Department of Fire and Disaster Protection, Emergency Dispatch Center, Freiburg, Germany; b Association of Dispatch Centers [Fachverband Leitstellen e.V.], Lemgo, Germany; c Department of Anesthesiology and Critical Care, Medical Center – University of Freiburg, Faculty of Medicine, University of Freiburg, Freiburg, Germany

## Abstract

Emergency call handling relies primarily on verbal information, which is often constrained by stress, language barriers, and limited situational awareness. This article describes the conceptual design and real-world implementation of video-assisted emergency calls (VEC) at the Freiburg dispatch center (Public Safety Answering Point, PSAP). Building on longstanding experience with innovative digital dispatch solutions, the Freiburg system integrates real-time video as a dispatcher-controlled, supplementary information channel, next to the voice call, within existing emergency call workflows. The approach is characterized by defined cases, standardized activation procedures, dedicated dispatcher training including psychological safeguards, and a security-focused IT architecture separated from critical dispatch infrastructure. The Freiburg model demonstrates a structured, pragmatic, and scalable pathway for responsibly integrating live video into emergency dispatch operations.

## Background

The quality of emergency call handling constitutes the foundation of every successful emergency response chain.^1^ While communication between callers and the dispatch center (Public Safety Answering Point, PSAP) has traditionally relied primarily on auditory information, the integration of real-time video transmission introduces a new dimension of situational assessment. The overarching objective is to use visual information to overcome barriers such as language difficulties or stress-induced miscommunication, to provide more precise guidance for first aid measures, and to optimize resource allocation through a more objective situational overview. The quality of emergency call interrogation represents the decisive first link in the emergency response chain.^2^ Until now, structured and qualified emergency call handling in the dispatch center relied exclusively on acoustic perception and the verbal descriptions provided by callers. In stressful situations, however, verbal communication often reaches its limits: language barriers, disorientation, or the emotional distress of callers can result in imprecise or misleading situational assessments.

In this context, the potential benefits of video support for lay rescuers have already been demonstrated in simulation studies.^3^ In general, live video is perceived as a helpful tool for situational assessment, although it is not considered suitable for all cases.^4^ One of the largest quantitative, prospective investigations into the impact of video-assisted emergency calls (VECs) reported a reduction in the number of emergency vehicles dispatched with the highest priority.^5^ Furthermore, it has been shown that video information can influence decision-making in dispatch centers. Linderoth et al. found that approximately half of VEC cases led to a revised situational assessment by the dispatcher.^6^ Research on the use, opportunities, and challenges of live video streaming in dispatch centers has largely focused on technical feasibility, user perceptions, and potential operational application.^7^, ^8^

Taken together, these findings suggest that integrating live video into dispatch operations offers a practical means to enhance the precision and effectiveness of emergency call assessment, bridging the informational gap between callers and dispatchers while complementing established acoustic-based protocols.

## Implementation example: Freiburg dispatch center (PSAP)

The Freiburg dispatch center serves an area of approximately 1531 km^2^ with around 510,000 residents and handles approximately 100,000 emergency calls per year, all received via the European emergency number 112, including around 450 out-of-hospital cardiac arrest (OHCA) cases. In the dispatch center, the triage protocol provided by SNA (*Softwareunterstützte Notruf Abfrage GmbH*) is used to support structured call interrogation and automated generation of incident categories and dispatch codes. Emergency responses are categorized into three priority levels: ambulance dispatch without lights and sirens (low priority), ambulance dispatch with lights and sirens (urgent), and combined dispatch of an ambulance and emergency physician with lights and sirens (life-threatening emergencies). The Freiburg dispatch center has gained recognition through several pioneering technological implementations, which have since seen nationwide adoption. The accurate localization of callers, one long-standing challenge in emergency services, was addressed in Freiburg as early as 2019 through the introduction of Advanced Mobile Location (AML) technology.^9^ This approach has since become standard practice in many dispatch centers across Europe. When a caller dials an emergency number, the mobile device automatically activates Wi-Fi, satellite-based positioning and all other location and position sensors at the beginning of the call, even if these functions were previously disabled. Once a location is determined, the coordinates are automatically transmitted to a central server, from which the dispatch center retrieves the data. Another innovation introduced at the Freiburg dispatch center in 2018 is a smartphone-based first responder alerting system using advanced algorithms, which has evolved into the largest network of its kind in Germany.^10^, ^11^

With the introduction of video communication in March 2026, a long-anticipated paradigm shift is now underway (Table 1). By transmitting real-time visual data from the incident scene directly to the dispatcher’s workstation, the information gap between caller and dispatch center can be substantially reduced. The motivation for this project is threefold:

• Precision: Objective assessment of injury patterns or damage severity enables more accurate dispatch decisions.
• Guidance: Layperson cardiopulmonary resuscitation (CPR) and first aid measures can be instructed more effectively through visual feedback.
• Safety: Improved situational awareness enhances the safety of responding emergency personnel by allowing earlier identification of potential hazards.

**Table 1:** Key factors for the development and operationalization of video-assisted dispatch, based on the implementation experience of the Freiburg Dispatch Center (Public Safety Answering Point, PSAP).

**Group**	**Factor**
Technical factors	Network coverage
	Smartphone hardware
	Smartphone operation system
	IT infrastructure
	Interoperability

Human factors	Caller
	Dispatcher
	Training
	Acceptance
	Psychological support

Operational factors	Use case selection
	Workflow integration

Regulatory factors	Legislation
	Data protection

Strategic factors	Funding
	Evaluation/quality assurance

## Criteria and capabilities for VEC activation

The initiation of a live video transmission is always situation-dependent and based on the dispatcher’s professional judgment. Suitable scenarios are:

Medical emergencies

• Telecommunicator cardiopulmonary resuscitation (T-CPR): Visual verification of correct compression depth and rate during chest compressions, correction of hand placement, and ensuring full chest recoil.^2^
• Dyspnea and level of consciousness: Assessment of respiratory rate, skin color (cyanosis), and patient responsiveness in cases of unclear symptom description.
• Severe trauma and other medical emergencies: Situational assessment to support triage and targeted instructions.

Firefighting and rescue operations

• Use of video emergency calls (VECs) for assessing fire development, traffic accidents, or hazardous situations.

Scene assessment and logistics

• Verification of the exact location in difficult terrain at unclear incident sites when GPS data are insufficient, as the callers may not be directly on site.
• Preparation for response units by transmitting images to the team already en route.

Unsuitable scenarios are suicidal intentions or mental health emergencies, ongoing acts of violence or threat situations and extreme visibility conditions (e.g. darkness). Also scenarios when the psychological well-being of staff is threatened.

## Workflow of the video emergency call (VEC)

**Fig. 1:**
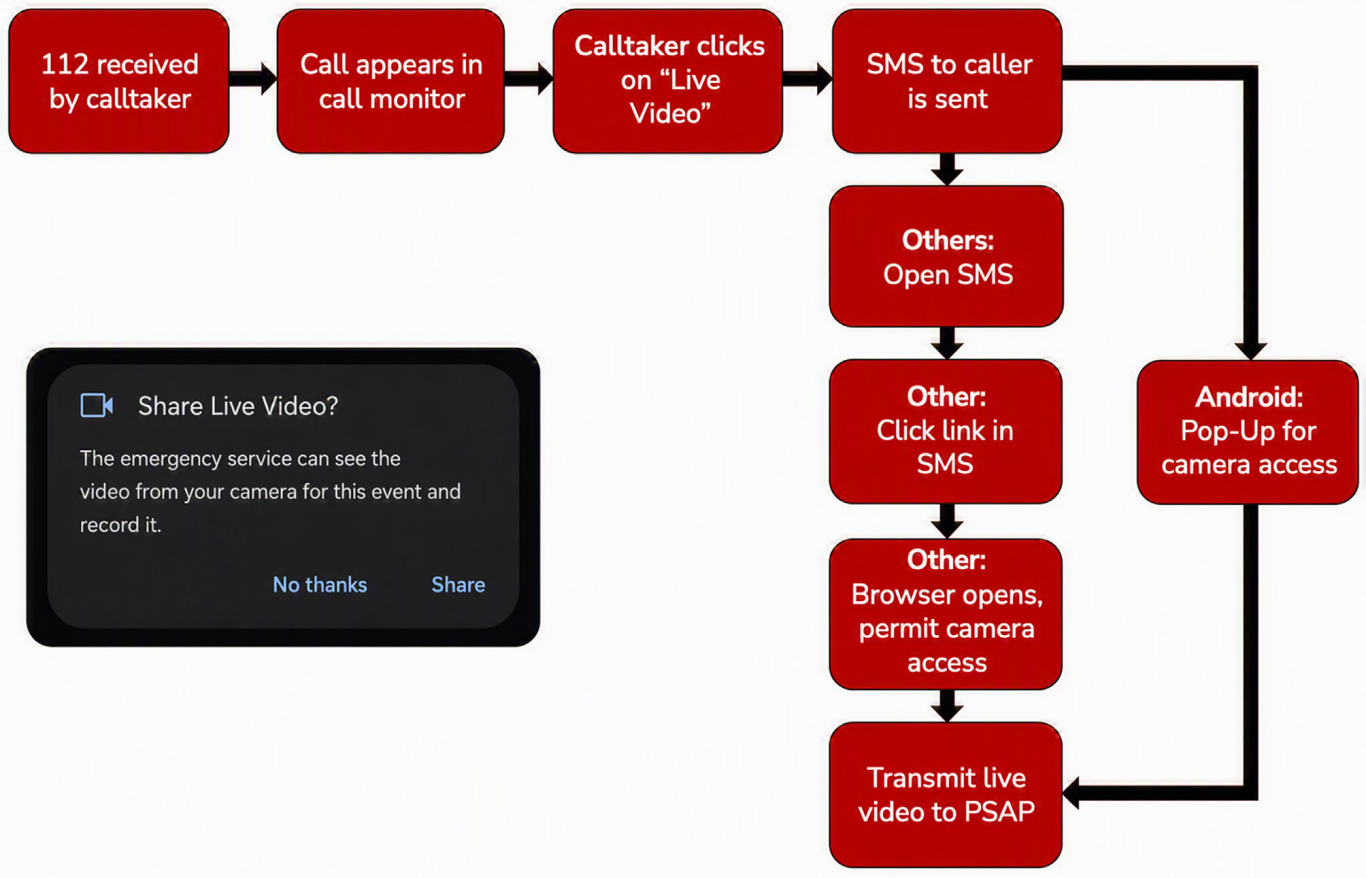
**Process flow of live video activation during a 112 emergency call**. After the emergency call is received and displayed in the call monitoring system, the call-taker initiates live video transmission by sending an SMS link to the caller. On Android devices, a system pop-up requests camera access, whereas on other devices the link is opened in a web browser and camera permissions are granted manually. Once permission is given, live video from the caller’s device is transmitted in real time to the dispatch center (Public Safety Answering Point, PSAP).

To ensure consistent quality and legally compliant handling, activation of live video communication follows a standardized, multi-step process (Fig. 1).

Once a dispatcher identifies a suitable use case, the following steps are initiated:

1. Verbal briefing: The dispatcher informs the caller: “I will now send you a link/request for a video connection so that I can better assess the situation. You must actively consent to camera access.”
2. Transmission of the access link: A system-generated SMS containing the access link is sent to the caller’s phone number directly from the dispatch system (Fig. 2).

**Fig. 2:**
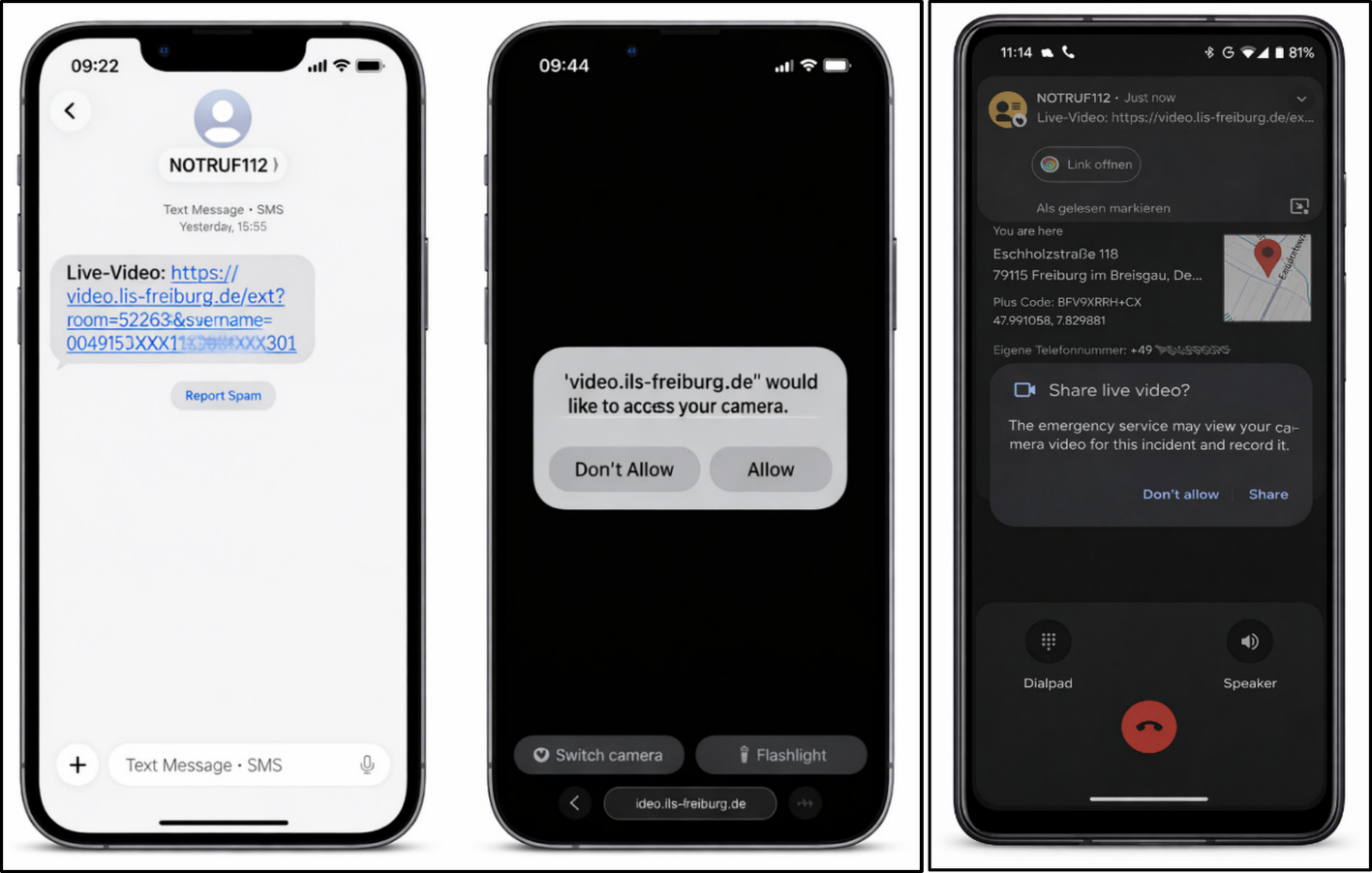
**Illustration of live video activation and guidance across different smartphone operating systems in an emergency call**. On iOS devices (*left*), the caller receives an SMS from the emergency service (“NOTRUF112”) containing a secure link to initiate a live video connection; upon opening the link, the device prompts for camera access, enabling real-time video transmission to support remote assessment and resuscitation guidance. On Android devices (*right*), the caller receives a system push notification with a secure link; opening the notification triggers a permission request to share live video. After consent is granted, real-time video is transmitted from the caller’s smartphone to the dispatch center (Public Safety Answering Point, PSAP), supporting dispatcher-assisted emergency care.
3. Technical activation: The caller opens the link (no app installation required) and confirms the privacy notice and camera access on their device. On Android devices supporting native live video emergency functionality, no SMS link is required. Instead, a pop-up notification appears directly on the screen (Fig. 2). After consent, the camera activates automatically and transmits the video stream to the dispatch center.

The video feed is displayed when the dispatcher activates the video room, while the audio connection remains on the primary telephony channel. The video stream serves as a supplementary information channel and does not transmit audio.

During the video session, the dispatcher provides clear instructions regarding camera positioning. An additional feature allows the dispatcher to capture screenshots for documentation or later handover to responding units. Care is taken to avoid prolonged capture of uninvolved third parties (e.g. bystanders).

The video connection is routinely terminated once the required information has been obtained or emergency units arrive on scene. It may also be ended if the visual content becomes psychologically overwhelming for the dispatcher or if filming poses a risk to the caller.

## Personnel and training

Dispatcher training in the federal state of Baden-Württemberg follows a structured, state-regulated curriculum combining theoretical modules with supervised practical training in the dispatch center. Personnel typically have a background in emergency services and undergo stepwise qualification as call takers and subsequently as dispatchers, ensuring standardized competencies in emergency call handling, decision-making, and resource allocation.

The introduction of video communication requires targeted preparation of dispatch center personnel. In addition to technical operation, emphasis is placed on tactical communication strategies and psychological self-protection. Building on their existing qualifications dispatchers in Freiburg received structured training specifically introducing the VEC system, including its background, functionality, and practical application. Before first use of the system, every dispatcher must complete a mandatory instruction consisting of the following modules: technology (e.g., system setup, activation procedures, troubleshooting), legal framework (e.g., data protection, consent, requirements), tactical application (e.g., use cases, integration into call workflows, situational assessment), and psychology (e.g., managing exposure to distressing visual content, optional termination of video transmission).

Unlike purely audio-based emergency call handling, video communication may expose dispatchers to unfiltered and potentially disturbing visual content.^12^ Therefore, structured psychosocial support is an integral component of the concept and includes:

• Training in vigilance management, i.e., consciously shifting visual focus away from distressing scenes when direct visual guidance is not required.
• The explicit right of dispatchers to terminate a video transmission at any time if overload is imminent, without this being considered a breach of duty.
• A peer-support system: Following particularly distressing video-assisted incidents, short debriefings with trained psychosocial emergency care personnel are available.

## IT architecture and data security

To prevent any interference with critical dispatch center infrastructure, the video solution is operated as a logically separated system from the computer-aided dispatch system and the communication management system.

• The video server (gateway) is located in a Demilitarized Zone (DMZ). There is no direct connection between the caller’s mobile device and the internal dispatch network.
• Dispatchers access the video stream via an internal video server acting as a proxy. Outbound connections from dispatcher workstations to the internet or other local network servers are restricted by strict firewall rules and are generally blocked.

No data are stored on the caller’s device during live video transmission. The system performs real-time streaming only; no recordings are generated by default.

Processing live video data from emergency situations entails a high risk to the rights and freedoms of affected individuals. Consequently, this concept is aligned with classical IT security objectives, adapted to the emergency services context: availability, integrity, confidentiality, and data minimization.

The processing of image and audio data within video-assisted emergency call handling follows a tiered legal framework, distinguishing between data relating to the caller and the incidental capture of third parties. The processing of personal data by the dispatch center is based on relevant provisions of the General Data Protection Regulation (GDPR). The specific legal mandate of dispatch centers in the federal state of Baden-Württemberg is established in regional emergency service and fire protection laws, as well as in the state data protection legislation.

## Summary and outlook

The implementation of real-time video communication at the Freiburg dispatch center represents a significant step toward digitally supported emergency medicine under stringent data protection and operational security standards. Designed as an adaptive and evolving system, the video emergency call framework continuously integrates technical, legal, and operational developments. Quality assurance is reinforced through structured training, regular debriefings, and systematic review of anonymized case examples, particularly to refine visual guidance in layperson resuscitation. Looking ahead, the Freiburg approach aims to strengthen precision, safety, and efficiency in emergency response while contributing to improved patient outcomes through the responsible integration of advanced telemedical tools into everyday dispatch practice. To further expand the evidence base and respond to calls for additional observational evidence on the use of video communication in emergency medical dispatch,^13^ we intend to address this through future investigations.

## Declarations

No personal data were processed for the purposes of this article.

## Consent for publication

Not applicable.

## Clinical trial number

Not applicable.

## **D**eclaration of generative AI and AI-assisted technologies in the manuscript preparation process

**Language check and picture translation:** The images included in this article were initially translated from German into English and generated using OpenAI’s ChatGPT, version GPT-5-mini (OpenAI, San Francisco, CA, USA) through its image translation functionality. All images have subsequently been carefully reviewed, validated, and, where necessary, edited by the authors to ensure accuracy, clinical correctness, and scientific integrity.

## CRediT authorship contribution statement

**Henning Schmidtpott:** Writing – original draft, Validation, Supervision, Project administration, Methodology, Data curation, Conceptualization. **Felix Reiser:** Writing – review & editing. **Erik Lachfeld:** Writing – review & editing, Visualization. **Sebastian Heinrich:** Writing – review & editing, Validation, Supervision. **Julian Ganter:** Writing – original draft, Validation.

## Declaration of competing interest

HS is member of the board of Association of Dispatch Centers (non-profit organization).

JG is member of the board of Region of Lifesavers (non-profit organization).

FR, EL and SB have no conflicts of interest.
